# Ferroptosis in Glioma Immune Microenvironment: Opportunity and Challenge

**DOI:** 10.3389/fonc.2022.917634

**Published:** 2022-06-27

**Authors:** Kaikai Wang, Junjie Wang, Jiahao Zhang, Anke Zhang, Yibo Liu, Jingyi Zhou, Xiaoyu Wang, Jianmin Zhang

**Affiliations:** ^1^ Department of Neurosurgery, The Second Affiliated Hospital, School of Medicine, Zhejiang University, Hangzhou, China; ^2^ Department of Neurosurgery, The Fourth Affiliated Hospital, International Institutes of Medicine, Zhejiang University School of Medicine, Yiwu, China; ^3^ Brain Research Institute, Zhejiang University, Hangzhou, China; ^4^ Collaborative Innovation Center for Brain Science, Zhejiang University, Hangzhou, China; ^5^ Clinical Research Center for Neurological Diseases of Zhejiang Province, Hangzhou, China

**Keywords:** glioma, ferroptosis, immune microenvironment, immunotherapy, GPX4

## Abstract

Glioma is the most common intracranial malignant tumor in adults and the 5-year survival rate of glioma patients is extremely poor, even in patients who received Stupp treatment after diagnosis and this forces us to explore more efficient clinical strategies. At this time, immunotherapy shows great potential in a variety of tumor clinical treatments, however, its clinical effect in glioma is limited because of tumor immune privilege which was induced by the glioma immunosuppressive microenvironment, so remodeling the immunosuppressive microenvironment is a practical way to eliminate glioma immunotherapy resistance. Recently, increasing studies have confirmed that ferroptosis, a new form of cell death, plays an important role in tumor progression and immune microenvironment and the crosstalk between ferroptosis and tumor immune microenvironment attracts much attention. This work summarizes the progress studies of ferroptosis in the glioma immune microenvironment.

## Introduction

Glioma is a threatening primary malignancy tumor in the central nervous system (1, 2), which is divided into grades I-IV according to WHO standard with glioblastoma (WHO grade IV) as the most malignant and common subtype (3). The standard therapy for glioma patients is the Stupp protocol, which consists of maximal safe surgical resection or a diagnostic biopsy, followed by concurrent chemoradiotherapy and then maintenance chemotherapy, where chemotherapy is comprised of temozolomide (4). Although glioblastoma (WHO IV) patients receive the most effective treatment/surgery with radiotherapy and chemotherapy after diagnosis (5, 6), the median survival time is only about 18 months (7), and that is mainly the result of a glioma infiltration boundary and/or the resistance of chemotherapy. Consequently, new therapeutic approaches for glioma are urgently needed (8).

Recently, immunotherapy represented by PD-1/PD-L1 and CTLA-4 has shown excellent clinical effects on numerous tumors such as melanoma and non-small cell lung cancer (9, 10), which have rekindled researchers’ faith in glioma treatment. Unfortunately, its effect is extremely limited in glioma and relevant clinical data show that it works on less than 10% of glioblastoma patients (11) An increasing number of studies have confirmed that it is a result of the glioma immunosuppressive microenvironment (12), therefore, the distruption of the immunosuppressive microenvironment and revision of the glioma from a ‘cold tumor’ to a ‘hot tumor’ is practical to relieve the glioma immunotherapy resistance (8).

The glioma immune microenvironment is composed of glioma cells, immune cells, cytokines and so on (12). Glioma cells can recruit numerous kinds of cell including immune cells that move to the niche by secreting cytokines (like TGF-β, GM-CSF) (13, 14) and then revise these cells into ‘tumor-friendly’ phenotypes (15). In this case, the recruited cells may serve as a physical barrier to prevent later immune cells from approaching and attacking the tumor cells Also, the recruited immune cells can also secrete cytokines (such as IL1-β, TGF-β) that continue to assimilate later recruited immune cells as ‘tumor-friendly’ phenotypes (16, 17). Under this “snowball” interaction, coupled with the unique central nervous system microenvironment, such as the blood-brain barrier (18) and hypoxia (19, 20), acidic tumor microenvironment (2, 21), tumor cells can escape immune surveillance (22) and eventually set the glioma immunosuppressive microenvironment (23, 24).

Ferroptosis is a form of regulated cell death driven by lipid peroxidation, a consequence of imbalance between cell metabolism and redox homeostasis (25). It is different from other cell death such as apoptosis, pyroptosis in morphology, biochemistry and gene (26). Its key process is phospholipids with polyunsaturated fatty acyl tails (PUFAs) are oxidized in an iron- or oxidoreductase- dependant way and ultimately induce cell death (27). Recently, researchers found that activating ferroptosis could improve temozolomide treatment effectiveness in GBM-bearing mice (28), and lonizing radiation could induce cell ferroptosis. The above means that ferroptosis is vital for glioma chemotherapy and radiotherapy (29).

## Overview of Ferroptosis and Potential Singling Pathway in Glioma

**Figure 1:**
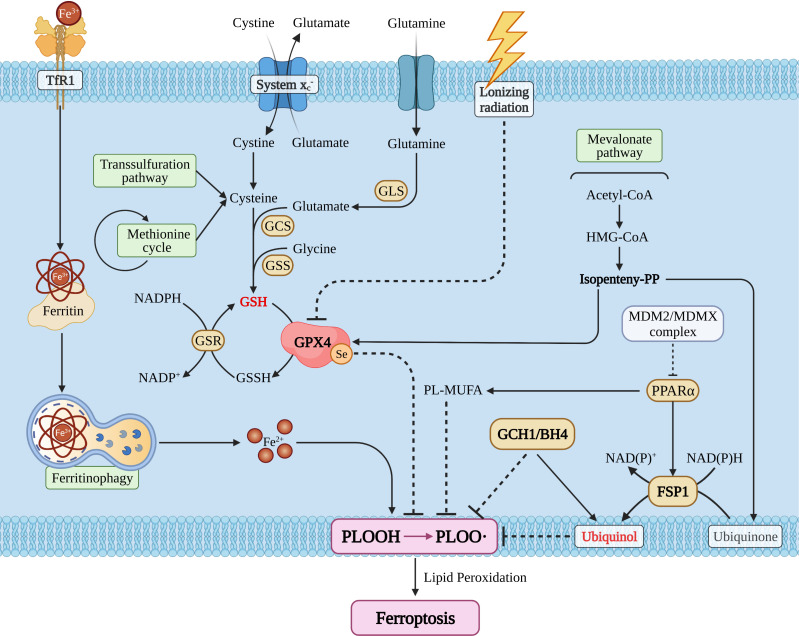
The snapshot of ferroptosis pathways. TfR1, transferrin receptor 1; GLS, glutaminase; GCS, glutamylcysteine synthetase; GSS, glutathione synthetase; GSH/GSSH, glutathione; GSR, glutathione S-reductase; GPX4, glutathione peroxidase 4; MDM2, mouse double minute 2; MDMX, mouse double minute 4; PPARα, peroxisome proliferator activated receptor alpha; FSP1, ferroptosis suppressor protein 1; GCH1, GTP cyclohydrolase 1; BH4, tetrahydrobiopterin; PL, phospholipid; MUFA, monounsaturated fatty acid.

The main characteristics of ferroptosis include: cell morphology (mitochondria crista, volume reduction, and increase of membrane density); cellular composition [cellular ROS is elevated and lipid peroxidation is significantly increased (27)]. Meanwhile, the intracellular pool of antioxidant executor (GSH or/and glutathione peroxidase 4) was shrunk, and phospholipid peroxide (PLOOH) is the executive driver of ferroptosis (26, 27). With step by step studies, researchers found that ferroptosis could be regulated by a variety of ways including redox homeostasis (30), iron metabolism (31), mitochondrial activity (32), metabolism of amino acids, lipids, and glucose (33). Ferroptosis pathways can be broadly divided into glutathione peroxidase 4 (GPX4) -dependent and -independent pathways (25, 26) (**Figure 1**).

### GPX4 Dependent Ferroptosis Pathway

Glutathione peroxidase 4 (GPX4), also known as phospholipid hydrogen peroxide glutathione peroxidase (PHGPx), is a selenoprotein required for peroxidized phospholipids (34). Cystine/glutamic acid reverse transporter (system $x_{\text{c}}^{-}$ ) is an upstream regulator (25, 35) and its dysfunction can increase glutamic acid levels and reduce cystine levels (36), which in turn leads to the exhaustion of the intracellular pool of glutathione (GSH), the main reducing substance of human body (37). Subsequently, this causes GPX4 reduction (27), then induces more PUFAs to turn to PLOOH and eventually induces ferroptosis (25). Besides, lonizing radiation also could regulate GPX4 activity directly and then shape ferroptosis (38).

System $x_{\text{c}}^{-}$ plays an important role in GPX4 relative pathway, whether the system $x_{\text{c}}^{-}$ dysfunction could result in the pool of GSH and GPX4 shrinking (35), and then gives birth to intracellular PLOOH explode and ultimately induces ferroptosis (26, 33). The monitors regulating system $x_{\text{c}}^{-}$ are SLC7A11 (39), SLC3A2 (40), NRF2 (41) and so on (42). Stephanie demonstrated that SLC7A11 expression is associated with seizures and predicts poor survival in patients with malignant glioma (43) Ju et al. proved that NRF2 is a potential prognostic biomarker and is correlated with immune infiltration in the brain’s lower grade glioma (44). Long et al. found that dysregulation of system $x_{\text{c}}^{-}$ enhances Treg function that promotes VEGF blockade resistance in glioblastoma (45). The above indicates that system $x_{\text{c}}^{-}$ should be a key hub between ferroptosis and the glioma immune-microenvironment.

Cystine metabolism is a vital segment in the GPX4-dependent ferroptosis pathway and the main factors affecting cystine metabolism include the transsulfuration pathway and/or the methionine cycle (46). As a vital brick for GSH synthesis, cystine plays a key role in glioma progression, Liu et al. confirmed that methionine and cystine double deprivation stress suppresses glioma proliferation by inducing reactive oxygen species (ROS) and autophagy (47), Wang et al. demonstrated that methionine deprivation can reset numerous immune pathways such as macrophages, T cell activation pathways in glioma (48), as cystine and methionine are all in methionine cycle (49), and there should be cystine/methionine-ferroptosis-immunity related pathways. Simultaneously, glioma cells can selectively uptake methionine, cysteine, and serine (47, 50, 51), so other cells will uptake or store less of these amino acids than glioma cells, which limits the production of cysteine and GSH. It remains to be determined whether it would induce other cells to include immune cells more sensitive to ferroptosis than glioma cells and whether DNA/RNA methylation is vital for glioma escape ferroptosis, as methionine is the major methyl donor (52, 53). Unfortunately, the researchers did not conduct this corresponding work.

In addition, the mevalonate pathway also participated in GPX4 activity regulation and isopentenyl pyrophosphate was the core factor regulating the transcription efficiency of GPX4 (54). E. Cimini et al. has confirmed that zoledronic acid, an aminobisphosphonate drug, can inhibit glioma cell proliferation by interfering with mevalonate pathway of Vγ2 T-cells (55). Deven found that LXRβ knockdown decreased cell cycle progression, cell survival, and decreased feedback repression of the mevalonate pathway in densely-plated glioma cells. LXRβ regulates the expression of immune response gene sets and lipids known to be involved in immune modulation (56) and these works imply that targeting the mevalonate pathway could disturb ferroptosis and immunity in glioma.

Currently, researchers have demonstrated that lonizing radiation could consume GSH, inhibit GPX4 activity, and induce ferroptosis (25, 54), and they denote that ferroptosis should be essential for glioma treatment because radiotherapy is an important part of Stupp strategy (8). Zhang et al. revealed that inhibition of TAZ contributes to radiation-induced senescence and growth arrest in glioma, and immune-related genes are specifically affected as the long-term effect (57). However, we did not know whether ferroptosis cells would act as or release cytokines that induce glioma cells to adapt to radiotherapy resistance.

### GPX4 Independent Ferroptosis Pathway

Although GPX4 is the core molecule of ferroptosis, we have now found other pathways that influence PLOOH synthesis and ferroptosis (25, 26).

The first is the ferroptosis inhibition protein 1(FSP1) (58–60), which can reduce the mevalonate pathway produced ubiquinone translate to ubiquinol, suppress production of PLOOH, and eventually inhibit ferroptosis (58). Furthermore, FSP1 could also be activated by the MDM2/MDMX-PPARα axis (25, 61), and in addition to activating FSP1 functions, PPARα also regulates the conversion of PL-MUFA to PLOOH by ACSL3-mediated MUFA way. It has been reported that FSP1 can protect cells from ferroptosis which is induced by GPX4 inhibition/knockout (26). Zou et al. demonstrated that TGF−β1 increases FSP1 expression in human bronchial epithelial cells (62), as TGF−β1 is an important cytokine that can be secreted by glioma or/and immune cell (17, 63, 64), and it means that FSP1 could be a nexus between glioma or/and immune cell ferroptosis.

A critical factor in inducing ferroptosis is the imbalance of intracellular iron metabolism which could cause iron overload. Through the specific receptor TFR1 (transferrin receptor 1), circulating iron (Fe^3+^) can be imported into the cell and stored mostly within ferritin (Fe^3+^), changing to cytoplasmic iron (65). A small pool of cytoplasmic free Fe^2+^ could directly catalyze the formation of free radical formation *via* the Fenton Reaction where changes of ferritin expression levels affect the homeostasis of iron metabolism by altering the intracellular free and redox active iron pool. Researchers have reported that the overexpression of NCOA4 reinforces the degradation of ferritin, which releases excessive cytoplasmic free Fe^2+^ and subsequently, promotes ferroptosis (66).

As a “double-edged sword”, autophagy is crucial in glioma progress (67, 68) due to the unbridled proliferation tumor cells that require a large amount of nutrients. Also, an appropriate level of autophagy is conducive to ensure necessary cellular function, as their own bricks, could be reused but excessive levels of autophagy will induce cell ‘self-digestion’ and eventually induce glioma death (69). Recent studies have proven that autophagy can participate in ferroptosis and the main progress is called ferritinophagy (25), and the key interaction hub of these two pathways is NCOA4 and FTH1. Zhang et al. confirmed that COPZ1 is the key molecule that mediates autophagy-dependent ferroptosis in glioma (70). Meanwhile, autophagy is essential for immune cell proliferation and function, and Enyong confirmed that autophagy-dependent ferroptosis drives tumor-associated macrophage polarization *via* the release and uptake of the oncogenic KRAS protein (71). Sun et al. confirmed that autophagy-dependent ferroptosis-related signature is closely associated with the prognosis and tumor immune escape of patients with glioma (72). Therefore, we recognize autophagy should be one of the hubs between ferroptosis and immunity in glioma.

Moreover, researchers have also confirmed that GTP cyclohydrase 1 (GCH1) inhibits the production of PLOOH through its metabolite BH2/4. Meanwhile, BH4 could also reduce PLOOH pool by regulating the production of Ubiquinol (26). Anh proved that the GCH1 knockdown with short hairpin RNA led to GBM cell growth inhibition and reduced self-renewal in association with decreased CD44 expression (73). Yan et al. showed that blocking CD44 inhibited glioma cell proliferation by regulating autophagy (67) and this means GCH1 could induce glioma cell ferroptosis and influence immunity by autophagy.

Furthermore, AMPK associated energy stress and Hippo pathways are all associated with ferroptosis by regulating the PLOOH pool (25, 26), and these factors are also vital for the glioma immunosuppressive microenvironment (GIME) and glioma proliferation (24).

## Ferroptosis and Immune Microenvironment

**Figure 2:**
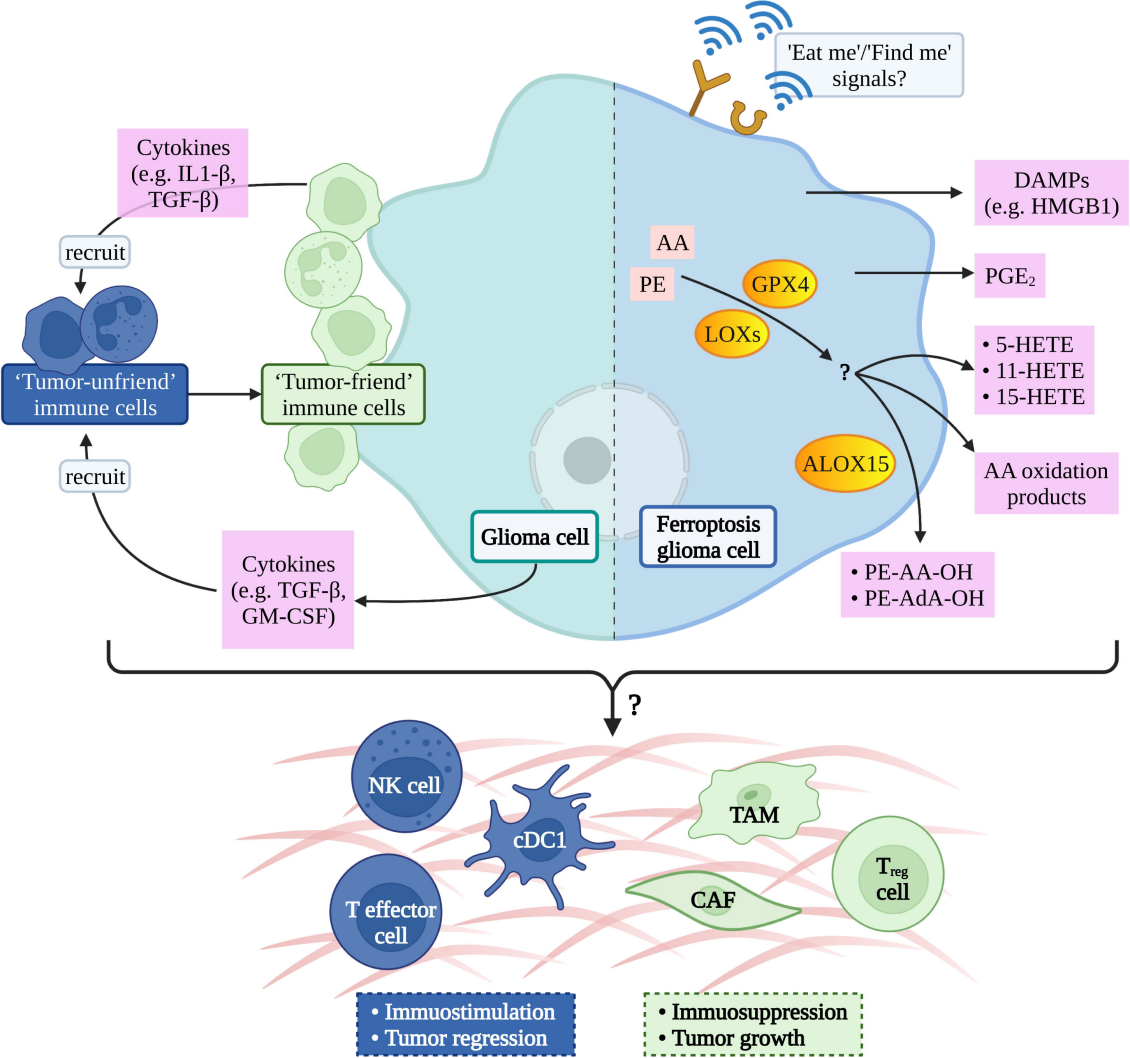
Possible ferroptotic signals in glioma immune-microenvironment. AA, arachidonic acid; PE, phosphatidylethanolamine; GPX4, glutathione peroxidase 4; LOXs, lipoxygenases; ALOX15, arachidonate lipoxygenase 15; DAMPs, damage-associated molecular patterns; HMGB1, high mobility group box-1; PGE2, prostaglandin E2; HETE, hydroxy eicosatetraenoic acid; AdA, adrenic acid; IL1, interleukin-1; TGF, transforming growth factor; GM-CSF, granulocyte-macrophage colony-stimulating factor; NK, natural killer; cDC1, conventional type 1 dendritic cell; TAM, tumor- associated macrophage; CAF, cancer-associated fibroblast; T reg cell, regulatory T cell.

Inducing tumor cell death is one of the effective methods to treat cancer, so inducing cancer cell ferroptosis is a feasible way for glioma treatment (34). Dead cells can release a series of “find me” and “eat me” signals for immune cells to locate, migrate, and clean dead cells which is confirmed by the phenomenon that ferroptosis tumor cells can be effectively engulfed by macrophages *in vitro (* 74). The calreticulin (CRT), a soluble ER-associated chaperone, is one of the ferroptosis-mediated proteins which regulate the tumor microenvironment. Ferroptosis facilitates the translocation of CRT to expose it on the surface of tumor cells, where CRT could serve as a potent “eat-me” signal and induce a robust antitumor immune response (75). However, the signal communication between ferroptosis glioma cells and surrounding immune cells is not clear (76) (**Figure 2**).

The potential signal is the arachidonic acid (AA) oxidation product released by ferroptosis cells therefore, it has been hypothesized that lipoxygenases (LOXs) can not only induce the PUFAs production but also promote ferroptosis cells to release immune signals and regulate tumor immunity (26). A study has shown that ferroptosis cells can release eicosanoids (5-HETE, 11-HETE, 15-HETE, etc.) when GPX4 was suppressed. Contrarily, ferroptosis cells reduce the production of pro-inflammatory lipids when GPX4 activity was increasing, afterwards inhibiting the production of TNF and IL-1 β by the NF-kB pathway (77). A liposome analysis of ferroptosis cells found that the accumulation of oxygenated AA-containing phosphatidylethanolamine species was associated with ALOX15 (78), which can shape adaptive immune response by inhibiting dendritic cell maturation and T cell helper cell 17 (TH17) differentiation *via* activating transcription factor NRF2 (79).

Prostaglandin E2 (PGE2) is considered to be one of the important immunosuppressive factors and it can be released after most death cells (26, 80) and then disturb immune cells mainly in the following ways: 1. directly inhibit NK, cytotoxic T cell clean function (81, 82), 2. inhibit the infiltration of Conventional Type 1 dendritic cell (cDC1) into tumor niche *via* inhibiting the secretion of CCL5 and XCL1 by NK cells (83), and 3. inhibit cDC1-dependent CD8^+^ T cell-mediated immune response (84). Yoshiteru proved that inhibition of macrophagic PGE2 synthesis is an effective treatment for the induction of anti-glioma immune response (85).

Recent studies have confirmed that GPX4 activity is associated with chronic inflammation (26), and current studies have confirmed that glioma progression is related to chronic inflammation (86). Moreover, Xu et al. demonstrated that GPX4 is crucial for protecting activated Treg cells from lipid peroxidation and ferroptosis and offered a potential therapeutic strategy to improve cancer treatment (87). All of the above has to remind us that GPX4 may be a hub to connect ferroptosis and inflammation/immune in glioma.

In addition to releasing lipid mediators, ferroptosis cells can also release HMGB1 in an autophagy-dependent manner (88). HMGB1 belongs to DAMPs and is one of the key elements for tumor cell immunogenicity, as it will bind to its receptor and activate the immune system once it is released outside of cell (89). Wen et al. confirmed that RAGE is essential for HMGB1 mediated TNF releasing in macrophage when they respond to ferroptosis cells (90). Lowenstein et al. considered that HMGB1-activated dendritic cells, loaded with glioma antigens, migrate to cervical lymph nodes to stimulate a systemic CD8^+^ T cells cytotoxic immune response against glioma and induce immunological memory (91).

In addition to the above cytokines, there are other cytokines worth exploring (76). Although researchers believe that the cytokines are critical for the “crosstalk” between ferroptosis cells and immune cells, the mechanism remains unclear. Additionally, attention should also be paid to off-target effects of ferroptosis induction (92).

## Challenges of Ferroptosis in GIME

The glioma immunosuppressive microenvironment (GIME) is the main reason for poor efficacy of immunotherapy in glioma (8, 22, 23). The rapid proliferation of glioma causes an arduous microenvironment such as acidity, limited of nutrients, and oxygen (47, 93, 94). In this circumstance, immune cells will betray, retreat, or die as they cannot adapt (95, 96) but glioma can adapt to this harsh microenvironment due to their own tremendous plasticity (97, 98). The blood-brain barrier can also hinder immune cells from migrating to the tumor (99, 100). Also, many of the inhibitory cytokines secreted by glioma (101) and inhibitory immune cells suppress the antitumor effect of immune cells (21, 102). In addition, glioma cells can also secrete numerous cytokines to trap immune cells as they present ‘non-tumor cells’ markers (95, 103) and then these “tricked” cells secrete cytokines and continue to later recruit immune cells (104). In this circumstance, glioma cells escape immune surveillance (22, 105)and we should take the above into account when considering glioma immunotherapy (106). Meanwhile, immunotherapy combination regimens (22), administration mode, and timing (107) can also influence the therapeutic efficacy. Currently, accruing studies demonstrate that ferroptosis is crucial for tumor progression and targeting ferroptosis maybe a latent way to remodel the tumor immune microenvironment (26, 27, 34). While we have already done a brief description above, we should also recognize the challenges.

Although ferroptosis does play a crucial role various tumor immune microenvironments, its own mechanism is still unclear (25, 26), which is reflected on the following aspects: 1. The exactly mechanism of PLOOH in ferroptosis is unclear. At present, although it is clear that PLOOH is the ultimate executor of ferroptosis, the exact mechanism of PLOOH inducing ferroptosis is unknown (26); 2. Ferroptosis studies lack a ‘gold standard’. Although we have made great progress in ferroptosis study (26), we have not yet found a relative “gold standard” like LC3, and P62 in autophagy (108) and researchers usually select one or more targets such as GPX4, P53, FTH1 (109–111) in a paper, even worse the targets just like scraped together, which has troubled the following researchers. 3. Ferroptosis shows a ‘double-edged sword’ role in diseases. It is easy to understand that ferroptosis plays different roles in different diseases such as the beneficial outcome of inducing ferroptosis in tumor cells is for disease (112), but inhibiting ferroptosis in stroke is beneficial to the prognosis (113). We hypothesize that ferroptosis may also play different roles in one disease, for example, and there may be tumor cells that choose to sacrifice themselves. Then, the secreted cytokines can make the surrounding tumor cells in a stress state and finally avoid ferroptosis (26). 4. What and how ferroptosis cells release signals after death and what are the functions of these signals (33, 71). 5. The crosstalk between ferroptosis and other forms of death is not clear (26, 27, 33). For cells that may suffer from different kinds of death at the same time (114), it is unknown how can they communicate with each other or whether ferroptosis works more or less in cell death. This is because we found cells suffer ferroptosis but cells still died after we used ferroptosis inhibitors in a proper dose. This means that ferroptosis can induce other kinds of cell death, and/or it plays a minor effect in cell death, or we use inhibitors after the ‘reversible point’ and once this threshold is exceeded, ferroptosis will be irreversible

Recently, we also found that ferroptosis is vital for tumor immunity such as macrophage phagocytosis (71) and T cell killing (115) but the “crosstalk” between ferroptosis and the glioma immunosuppressive microenvironment is not clear. Additional issues to be addressed are: 1. The signal interaction between ferroptosis cells and surrounding immune cells is not clear which is mainly manifested in the specific cytokines of ‘find me’ and ‘eat me’ released by ferroptosis cells (26). 2. Will the cytokines released by ferroptosis cells help other glioma cells escape immune surveillance by seducing or misleading immune cells (34, 88, 103)? 3. Whether GPX4-induced chronic inflammation engaged in glioma progression or outcome (116). 4. What is the role of ferroptosis in glioma immunotherapy tolerance? (95, 103).

## Conclusion

The clinicians and researchers are always trying to find new treatments for tumors and it is comforting that treatment methods such as immunotherapy and oncolytic virus have been found. Unfortunately, immunotherapy, which has shed light on numerous tumor treatments, does not always work regarding glioma. Increasing research demonstrates that this is result of the glioma immunosuppressive microenvironment, so researchers are searching for an antidote for remodeling GIME. Ferroptosis, a new form of cell death, plays an important role in glioma cell and immune cell. The exactl mechanism is unclear and multipley works demonstrate that it is deserved to explore its role in GIME and how to regulate ferroptosis for glioma therapy. Although there are still many obstacles in the cognition of crosstalk between ferroptosis and GIME, we believe we will address this with further studies and new technologies, such single cell sequencing and spatial transcriptomics. This will not only improve our understanding of ferroptosis and GIME but also provide a new solution for glioma immunotherapy resistance, a new breakthrough point for glioma treatment.

## Author Contributions

KW proposed the research. KW, JW, and JHZ wrote the manuscript and finalized the paper. AZ, YL, and JYZ both reviewed the literature and collected references. XW and JMZ reviewed the literature. All authors contributed to the article and approved the submitted manuscript.

## Funding

This work was funded by National Natural Science Foundation of China (No. 82002634, 81171096 and 81371433).

## Conflict of Interest

The authors declare that the research was conducted in the absence of any commercial or financial relationships that could be construed as a potential conflict of interest.

## Publisher’s Note

All claims expressed in this article are solely those of the authors and do not necessarily represent those of their affiliated organizations, or those of the publisher, the editors and the reviewers. Any product that may be evaluated in this article, or claim that may be made by its manufacturer, is not guaranteed or endorsed by the publisher.
